# Relationship Between 8-iso-prostaglandin-F_2α_ and Predicted 10-Year Cardiovascular Risk in Hypertensive Patients

**DOI:** 10.3390/life15030401

**Published:** 2025-03-04

**Authors:** Giulio Geraci, Alessandra Sorce, Luca Zanoli, Giuseppe Cuttone, Vincenzo Calabrese, Francesco Pallotti, Valentina Paternò, Pietro Ferrara, Ligia J. Dominguez, Riccardo Polosa, Jacob George, Giuseppe Mulè, Caterina Carollo

**Affiliations:** 1 Department of Medicine and Surgery, Kore University of Enna, 94100 Enna, Italy; giulio.geraci@unikore.it (G.G.); giuseppe.cuttone@unikore.it (G.C.); vincenzo.calabrese@unikore.it (V.C.); francesco.pallotti@unikore.it (F.P.); ligia.dominguez@unikore.it (L.J.D.); polosa@unict.it (R.P.); 2 Unit of Nephrology and Dialysis, Hypertension Excellence Centre, Department of Health Promotion, Mother and Child Care, Internal Medicine and Medical Specialties (PROMISE), University of Palermo, 90133 Palermo, Italy; alessandra.sorce@community.unipa.it (A.S.); giuseppe.mule@unipa.it (G.M.); 3 Nephrology, Department of Clinical and Experimental Medicine, University of Catania, 95124 Catania, Italy; luca.zanoli@unict.it; 4 Operative Unit of Diabetology, Umberto I Hospital, Provincial Health Authority (ASP) of Enna, 94100 Enna, Italy; 5 Center for Public Health Research, University of Milan–Bicocca, 20900 Monza, Italy; pietro.ferrara@unimib.it; 6 Laboratory of Public Health, IRCCS Istituto Auxologico Italiano, 20149 Milan, Italy; 7 Center of Excellence for the Acceleration of Harm Reduction, University of Catania, 95124 Catania, Italy; 8 Cardiovascular Medicine and Therapeutics, University of Dundee Medical School, Ninewells Hospital, Dundee DD1 9SY, UK; j.george@dundee.ac.uk

**Keywords:** oxidative stress, 8-iso-prostaglandin-F_2α_ cardiovascular risk, hypertension, chronic kidney disease, inflammation, prevention, prognosis

## Abstract

Background: 8-iso-prostaglandin-F_2α_ (8-iso-PGF_2α_) is a recognized marker of oxidative stress. Previous studies suggested that 8-iso-PGF_2α_ plays an important role in the pathogenesis of hypertension and cardiovascular (CV) diseases. However, limited data exist on the prognostic role of 8-iso-PGF_2α_ in hypertensive patients undergoing primary prevention. The aim of this study was to assess the relationship between 8-iso-PGF_2α_ and 10-year CV risk, as predicted by validated equations in hypertension patients without CV diseases. Materials and methods: A total of 432 individuals aged 40–75 years were enrolled. Plasma 8-iso-PGF_2α_ was assessed through the ELISA method. CV risk was calculated by using the Framingham Risk Score (Fr-S) and the Atherosclerosis Cardiovascular Disease Risk Score (ASCVD-S). Low, moderate, or high CV risks were defined according to validated cutoffs. Results: Individuals with higher CV risk had significantly greater 8-iso-PGF_2α_ values compared to those with low or moderate CV risk (*p* < 0.001). 8-iso-PGF_2α_ correlated strongly with Fr-S and ASCVD-S in the entire population and in patients with normal renal function (all *p* < 0.001) but not in patients with eGFR < 60 mL/min/1.73 m^2^. These associations remained significant after adjustment for traditional factors included in the CV risk equations in the overall population and in patients with normal renal function. The 8-iso-PGF_2α_ cutoffs that best distinguished patients with high CV risk were 310 pg/mL for Fr-S and 264 pg/mL for ASCVD-S in the overall population, with significant differences between the groups divided by eGFR (all *p* < 0.001). Conclusions: These findings highlight the potential utility of 8-iso-PGF_2α_ as a biomarker for refining cardiovascular risk stratification in hypertensive patients, particularly those with preserved renal function. Future studies should explore its prognostic value in longitudinal cohorts and assess its integration into clinical risk models to enhance early prevention strategies for cardiovascular disease.

## 1. Introduction

The 8-iso-prostaglandin-F_2α_ (8-iso-PGF_2α_) is a bioactive compound mainly formed in humans via the free radical-mediated peroxidation of arachidonic acid in membrane phospholipids [1]. It serves as a valuable biomarker of in vivo lipid oxidation and a surrogate indicator of increased reactive oxygen species production and reduced nitric oxide bioavailability. As such, it is considered a sensitive and specific index of oxidative stress [2,3]. Previous studies have demonstrated that 8-iso-PGF_2α_ induces vasoconstriction, platelet activation, and smooth muscle proliferation in blood vessels [4]. These effects contribute to the impairment of endothelium-mediated vasodilatation and promote a pro-thrombotic and pro-inflammatory state [3,4,5,6].

There is considerable evidence that F_2_-isoprostanes are involved in the pathogenesis of hypertension and atherosclerosis, and their important role in the development of cardiovascular diseases has also been described [7,8,9,10]. Oxidative stress contributes to the adverse effects of cardiovascular risk factor by inducing endothelial dysfunction and morphofunctional changes in microcirculation, both of which are strong predictors of organ damage and cardiovascular outcomes [5,6,11,12].

Over the years, several mathematical models have been developed to estimate global risk of cardiovascular events by integrating the partial information of each major risk factor. Among these, the Framingham Risk Score (Fr-S) and the Atherosclerosis Cardiovascular Disease Risk Score (ASCVD-S) have become widely adopted due to their simplicity and validation across diverse populations [13,14,15]. Developed from longitudinal trial data, these scores can estimate the 10-year risk of cardiovascular diseases, and their use is recommended to assess overall cardiovascular risk, providing a comprehensive approach to managing hypertensive patients [15,16,17].

The early detection of cardiovascular risk in hypertensive patients is crucial for implementing timely interventions that can reduce the progression of cardiovascular disease and associated complications. Identifying high-risk individuals at an early stage allows for targeted lifestyle modifications, optimized pharmacological treatment, and personalized preventive strategies. The use of reliable biomarkers, such as 8-iso-PGF_2α_, as a marker of in vivo oxidative stress, can enhance risk stratification by providing objective measures of oxidative stress, a key contributor to vascular dysfunction [1]. Integrating such biomarkers into routine clinical assessments may improve risk prediction, facilitate early therapeutic decisions, and ultimately contribute to the prevention of hypertension-related cardiovascular events [7,8,9,18].

In this context, 8-iso-PGF_2α_ emerges as a potential mediator between oxidative stress and cardiovascular risk assessment, potentially serving as a predictor of cardio-cerebrovascular diseases across diverse populations [7,8,9]. While some studies support this association, conflicting data remain [18]. For example, a systematic review by Zhang et al., summarizing findings from 20 studies, reported a significant correlation between elevated F_2_-isoprostanes levels in urine or blood and cardiovascular disease [9]. However, the authors emphasized the need for further research to clarify the role of F_2_-isoprostanes as a non-specific indicator of cardiovascular disease.

Given the limited evidence on the prognostic value of 8-iso-PGF_2α_, a specific F_2_-isoprostane, in hypertensive patients, particularly in the context of primary prevention, our study seeks to investigate its potential as a biomarker for predicting 10-year cardiovascular risk. Importantly, no prior research has explored the relationship between oxidative stress, as measured by 8-iso-PGF_2α_, and cardiovascular risk predictions derived from validated risk models in hypertensive patients. Addressing this gap, our study aims to provide new insights into the clinical relevance of oxidative stress markers in cardiovascular risk stratification.

## 2. Materials and Methods

### 2.1. Study Design and Population

This cross-sectional observational design was performed on 432 essential hypertensive patients selected from Caucasian patients consecutively attending the Nephrology and Hypertension Section of the University Hospital of Palermo, Italy, for specialist advice, between January 2024 and October 2024. Patients meeting the following criteria were excluded from this research (see Supplementary Figure S1):

− Aged <40 and >75 years old, to align with the application range of Fr-S and ASCVD-S;
− Renovascular, endocrine, or malignant hypertension or hypertension associated with obstructive sleep apnea syndrome, as described in detail in previous studies [19,20];
− Renal replacement therapy (transplanted or dialysis patients);
− Pharmacological treatment for cardiac rhythm or conduction abnormalities, in order to minimize potential confounders;
− Use of nonsteroidal or steroidal anti-inflammatory medications within 4 weeks before the start of the study.
− History of cerebrovascular disease, coronary heart disease, or symptomatic peripheral arterial disease;
− Hospitalization for CV cause in the previous 6 months;
− Major non-cardiovascular diseases (history of liver cirrhosis, chronic obstructive lung disease, or neoplasms).

The study protocol conformed to the ethical guidelines of the Declaration of Helsinki on ethical principles for medical research involving human subjects (REF) and was approved by the Local Review Board. Written informed consent was obtained from each patient.

### 2.2. Clinical and Laboratory Evaluation

Careful clinical history and physical examination were performed in all patients. Individuals who reported smoking cigarettes regularly during the past year were considered current smokers. Body weight and height were measured by a nurse, and body mass index (BMI) was calculated as body weight divided by squared height (kg/m^2^). Patients with a history of diabetes (or on treatment with antidiabetic drugs) or with fasting serum glucose levels of ≥126 mg/dL were considered diabetics. For individuals with fasting serum glucose levels of between 100 and 125 mg/dL, the diagnosis of diabetes was confirmed based on either glycated hemoglobin or 2 h plasma glucose during an oral glucose tolerance test. Clinic blood pressure (BP) was recorded by a doctor as the mean of three consecutive measurements obtained at 2 min intervals using a validated electronic oscillometric device (WatchBP Office, Microlife AG, Widnau, Switzerland), after 5 min of rest in a sitting position. According to the 2023 European Society of Hypertension/European Society of Cardiology guidelines, hypertension was defined as a BP ≥ 140/90 mmHg or treatment with antihypertensive drugs [17].

At 08:30 h on the day of the study, the overnight-fasted patients were placed in a supine position, and blood samples were obtained from an indwelling forearm venous catheter. Routine biochemical parameter determination was performed in all patients with standard techniques using an autoanalyzer (Boehringer Mannheim for Hitachi system 911, Mannheim, Germany). Low-density lipoprotein (LDL) cholesterol was calculated using the Friedewald formula. Estimated glomerular filtration rate (eGFR) was determined using the Chronic Kidney Disease Epidemiology Collaboration (CKD-EPI) equation. The 8-iso-PGF_2α_ was measured by a solid-phase, specific enzyme-linked immunosorbent assay (ELISA) using a commercial kit (Assay Design Inc., Ann Arbor, MI, USA), with particular attention to minimizing interference from other serum components. After centrifugation at 4 °C, blood samples were frozen at −80 °C and processed within 2 months of collection. According to the manufacturer’s recommendations, the test sensitivity was 16.3 pg/mL, and the inter-assay coefficient of variation was <9%. High-sensitivity C-reactive protein (CRP) was also measured using a commercially available ELISA kit (Diagnostic Biochem, London, Ontario, Canada), with a sensitivity of 10 ng/mL, an inter-assay coefficient of variation of <10%, and an intra-assay coefficient of variation of <8%.

Cardiovascular risk score: Past medical history, clinical data, and laboratory tests were collected in all patients to predict the 10-year risk of cardiovascular events using validated equations of Fr-S and ASCVD-S. These mathematical sex- and race-specific models provide an estimate of overall 10-year cardiovascular risk, and were, respectively, derived by the Framingham cohort study and pooled cohorts of participants from several large studies, including the Atherosclerosis Risk in Communities (ARIC) study, the Cardiovascular Health Study, and the Coronary Artery Risk Development in Young Adults (CARDIA) study. Based on specific cut-offs, the patients were classified into low CV risk (Fr-S < 10%; ASCVD < 7.5%), intermediate CV risk (Fr-S ≥ 10% and <20%; ASCVD ≥ 7.5% and <15%), or high CV risk (Fr-S ≥ 20%; ASCVD ≥ 15%) [15,16,17,18,19,20,21].

### 2.3. Statistical Analysis

Statistical analyses were performed using the IBM SPSS Statistics software package, version 23, for Macintosh (SPSS, Chicago, IL, USA). Statistical analysis was initially performed on the entire study population. Given the well-established link between 8-iso-PGF_2α_ and kidney function, as noted in previous studies [22,23], statistical analysis was subsequently conducted on two subgroups based on eGFR: ≥60 mL/min/1.73 m^2^ (*n* = 279) and <60 mL/min/1.73 m^2^ (*n* = 153). For further analyses, the population was divided into three groups according to validated 10-year cardiovascular risk cutoff values (10% and 20% for Fr-S; 7.5% and 15% for ASCVD-S) [15,16,17,18,19,20,21].

The normal distribution of continuous variables was assessed using the Kolmogorov–Smirnov test. Continuous variables were reported as means ± standard deviation (SD). Triglycerides, Fr-S, and ASCVD-S, which had skewed distributions, were log-transformed to satisfy distributional assumptions before applying parametric tests. These variables were presented as median and interquartile range (IQR). Categorical variables were expressed as percentages. Comparisons of continuous variables between groups were conducted using Student’s *t*-test for unpaired data or analysis of variance (ANOVA) with the Holm–Sidak test for multiple comparisons, as appropriate. For the categorical variables, comparisons were performed using the *χ*^2^ test, with the Monte Carlo method employed to compute exact two-tailed α-values.

Univariate regression analyses with Pearson’s correlation coefficients were used to examine the relationships between 8-iso-PGF_2α_ with Fr-S, ASCVD-S, and the other variables. Stepwise multivariate regression analyses were performed with Fr-S (or alternatively ASCVD-S) as the outcome variable. Covariates included: age, sex (0 = females; 1 = males), diabetes (0 = no; 1 = yes), current smoking habit (0 = no; 1 = yes), antihypertensive therapy (0 = no; 1 = yes), BMI, serum total cholesterol, HDL and LDL-cholesterol, clinic systolic BP, eGFR, and 8-iso-PGF_2α_. To further assess the influence of renal function, treated as continuous variable (eGFR), on the relationship between Fr-S (or alternatively ASCVD-S) and 8-iso-PGF_2α_, additional multivariate models were analyzed in the population divided into two groups based on eGFR values (≥60 mL/min/1.73 m^2^ or <60 mL/min/1.73 m^2^). A backward stepwise procedure was used in all analyses, with α equal to 0.15 as the cutoff for variable entry or removal. Collinearity was assessed by calculating the variance inflation factor (VIF): variables with VIF ≥ 2 were excluded from the models. The null hypothesis was rejected with a two-tailed *p*-value ≤ 0.05.

Receiver-operating characteristic (ROC) curves were built for the entire population and for the two groups divided by eGFR to evaluate the accuracy of 8-iso-PGF_2α_ in detecting a 10-year risk of cardiovascular disease ≥20% with Fr-S or ≥15% with ASCVD-S. The significance of differences between ROC curves was assessed using the Hanley and McNeil method. The null hypothesis was rejected at a *p*-value of ≤0.05.

## 3. Results

A total of 432 hypertensive patients were enrolled. The mean age of the overall study population was 60 ± 10 years; 59.0% were male and 35.4% had an eGFR of <60 mL/min/1.73 m^2^. Table 1 presents the characteristics of the overall study population and the two groups divided by eGFR.

Most of the individuals had a low cardiovascular risk, and none had a history of previous cardiovascular events. Patients with low eGFR had significantly higher values of 8-iso-PGF, Fr-S, and ASCVD-S compared to those with normal eGFR (all *p* < 0.001), while no significant differences in antihypertensive therapy were observed between groups.

**Figure 1:**
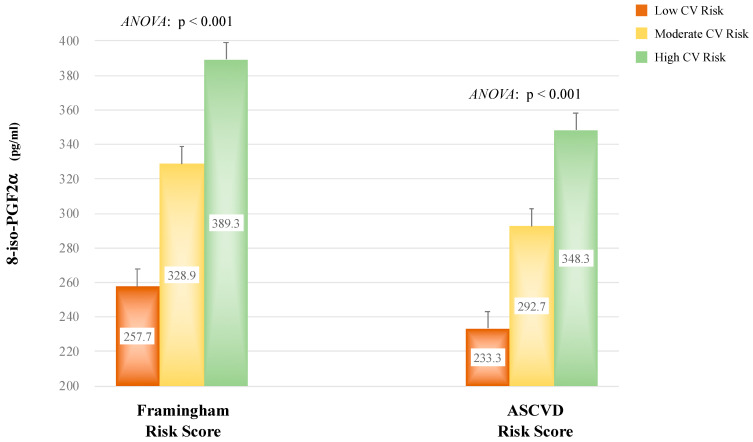
8-iso-Prostaglandin F_2α_ levels in patients with low, moderate, or high cardiovascular risk (CV) calculated by the Framingham Risk Score or Atherosclerotic Cardiovascular Disease Risk Score.

Subjects with higher cardiovascular risk had significantly greater values of 8-iso-PGF_2α_ compared to those with low or moderate cardiovascular risk (Figure 1).

The main univariate correlations of 8-iso-PGF_2α_, Fr-S, and ASCVD-S in the overall population are presented in Table 2.

**Figure 2:**
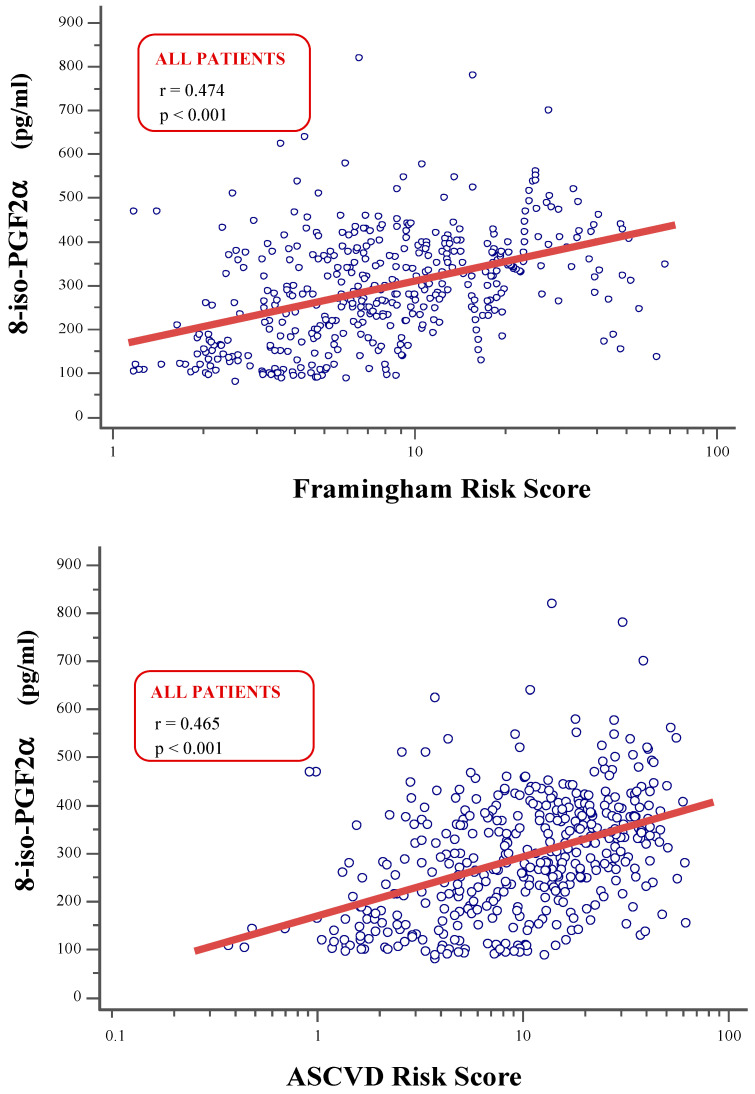
Univariate correlations between8-iso-Prostaglandin F_2α_ levels nd Framingham Risk Score (**upper plot**) or Atherosclerotic Cardiovascular Disease Risk Score (**lower plot**) in the entire study population.

8-iso-PGF_2α_ was significantly associated with the variables included in the equations used to predict 10-year cardiovascular risk. Furthermore, 8-iso-PGF_2α_ showed a strong correlation with both Fr-S or ASCVD-S (all *p* < 0.001) (Figure 2), and these relationships remained significant after adjustment for eGFR values (r = 0.361 and *p* < 0.001 with Fr-S; r = 0.306 and *p* < 0.001 with ASCVD-S). When these relationships were assessed separately in the two groups of patients divided by eGFR, 8-iso-PGF_2α_ was significantly associated with Fr-S and ASCVD-S only in subjects with eGFR ≥ 60 mL/min/1.73 m^2^ (respectively r = 0.667 and r = 0.580; all *p* < 0.001). In contrast, these correlations were not observed in subjects with lower eGFR< 60 mL/min/1.73 m^2^.

At the multivariate analyses in the overall population, 8-iso-PGF_2α_ was significantly associated with Fr-S (or alternatively ASCVD-S) independently of other covariates, including eGFR and variables used to calculate the 10-year cardiovascular risk (Table 3).

Additional multivariate models were constructed for subgroups with eGFR ≥ 60 mL/min/1.73 m^2^ and <60 mL/min/1.73 m^2^, and 8-iso-PGF_2α_ was independently associated with Fr-S and ASCVD-S only in individuals with eGFR ≥ 60 mL/min/1.73 m^2^ (all *p* < 0.001), whereas no significant relationship was observed in individuals with renal impairment.

**Figure 3:**
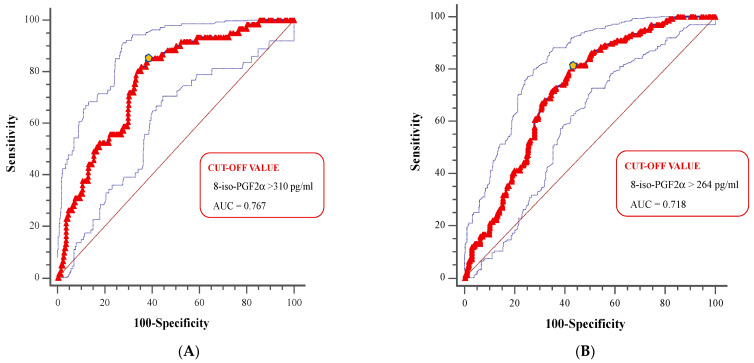
Receiver operating characteristic (ROC) curves of 8-iso-Prostaglandin F_2α_ levels for the detection of high cardiovascular risk calculated by Framingham Risk Score or Atherosclerotic Cardiovascular Disease Risk Score in the overall study population (**A**,**B**).

The ROC curves created to assess the global accuracy of 8-iso-PGF_2α_ in detecting patients with high cardiovascular risk (Fr-S ≥ 20%; ASCVD-S ≥ 15%) are shown in Figure 3. The 8-iso-PGF_2α_ cutoffs that best distinguished patients with high cardiovascular risk were 310 pg/mL for Fr-S (AUC: 0.767) and 264 pg/mL for ASCVD-S (AUC: 0.718) (Figure 3A,B).

When ROC curves were compared in patients stratified by eGFR, higher AUC values were observed in patients with eGFR ≥ 60 mL/min/1.73 m^2^ compared to those with lower eGFR, with significant differences (all *p* < 0.001; Figure 3A,B).

When ROC curves were compared in patients stratified by eGFR, higher AUC values were observed in patients with eGFR ≥ 60 mL/min/1.73 m^2^ compared to those with lower eGFR, with significant differences (all *p* < 0.001).

Patients with higher predicted cardiovascular risk had significantly elevated levels of 8-iso-PGF_2α_, which strongly correlated with the two risk scores considered, particularly in those with normal renal function. The association remained significant after adjusting for traditional risk factors but was not observed in patients with impaired kidney function, suggesting a potential influence of renal status on oxidative stress markers.

## 4. Discussion

A key finding of our study is that 8-iso-PGF_2α_, a reliable marker of oxidative stress, is independently associated with 10-year cardiovascular risk, as predicted by validated equations in hypertensive patients without overt cardiovascular disease. There is experimental evidence that oxidative stress contributes to the pathogenesis of hypertension [3,11,12], and previous studies have investigated the potential role of 8-iso-PGF_2α_ in the process of atherosclerosis and cardiovascular diseases [7,8,9,10,22]. Minuz et al. demonstrated the increased urinary excretion of 8-iso-PGF_2α_ in 75 hypertensive individuals compared to 75 pair-matched healthy controls [23], and other authors similarly found elevated urinary F_2_-isoprastanes in hypertensive patients and individuals at risk for future cardiovascular events [4,10]. Cottone et al. observed higher serum levels of 8-iso-PGF_2α_ in individuals with essential hypertension compared to healthy controls, confirming that oxidative stress is increased in this population [3]. High levels of F_2_-isoprostanes have also be proposed as a biomarker of cardiovascular disease, and the role of 8-iso-PGF_2α_ in cardiovascular events has also been investigated by several authors. In a large general population study, Keaney et al. reported that urinary 8-epi-PGF_2α_ levels were associated with previous cardiovascular diseases [24]: in this study, approximately 13% of participants had a history of prior cardiovascular events, and only one-third of patients had hypertension. In contrast, in our study, none of participants had overt cardiovascular diseases, and most had low cardiovascular risk, despite all being hypertensive.

The predictive role of urinary 8-iso-PGF_2α_ in cardiovascular mortality was also demonstrated in a case–cohort study of postmenopausal women [25], with similar findings reported in different populations [7,8,26]. However, most of these studies included patients with acute cardiovascular events, and the association between 8-iso-PGF_2α_ and cardiovascular events was not adjusted for the full set of Fr-S covariates. In our work, by contrast, 8-iso-PGF_2α_ was significantly associated with Fr-S or ASCVD-S even after adjustment for all variables used to predict 10-year cardiovascular risk.

Another relevant finding of our study is that 8-iso-PGF_2α_ was independently associated with cardiovascular risk only in subjects with eGFR ≥ 60 mL/min/1.73 m^2^, whereas this relationship was not observed in patients with lower eGFR. Previous studies consistently demonstrated enhanced oxidative stress in experimental and clinical renal injury [22,27,28]. Roberts and Morrow showed that the increased 8-iso-PGF_2α_ in renal dysfunction was due to a true excess in oxidative stress rather than an impaired metabolism or clearance of 8-iso-PGF_2α_ itself [29]. Cottone et al. observed a strong negative correlation between 8-iso-PGF_2α_ and eGFR in hypertensive individuals [22], and we confirmed these results by showing an inverse correlation between these variables (see Table 2), as well as higher 8-iso-PGF_2α_ values in individuals with normal renal function compared to those with renal impairment (*p* < 0.001, see Table 1). However, we did not find an association between 8-iso-PGF_2α_ and cardiovascular risk in patients with eGFR < 60 mL/min/1.73 m^2^. Oxidative stress is likely to represent an early stage in the development of atherosclerotic damage, serving as a primum movens that triggers endothelial dysfunction and contributes to the formation of atherosclerotic plaques [10,12,28]. Patients with renal impairment are likely to have widespread subclinical vascular damage, with other factors contributing to an elevated cardiovascular risk [19,30,31,32]. In line with this, several studies have highlighted that CKD individuals exhibit accelerated atherosclerosis [30,33,34] and greater organ damage compared to non-CKD individuals [34,35,36,37]. It is plausible that the vascular modifications in CKD patients are so advanced that they become independent of oxidative stress. Therefore, the lack of association between 8-iso-PGF_2α_ and cardiovascular risk in patients with eGFR < 60 mL/min/1.73 m^2^ may reflect advanced vascular damage rather than a true absence of oxidative stress contribution, thereby diminishing its prognostic significance. Further research is needed to explore these mechanisms and to better understand the underlying processes in this population.

To the best of our knowledge, this is the first study to report on the association between 8-iso-PGF_2α_ and predicted cardiovascular risk in hypertensive patients without overt cardiovascular disease. Some pathophysiological mechanisms could be hypothesized to explain this relationship. The process of atherosclerosis, which underlies cardiovascular events, recognizes sequential steps, from endothelial dysfunction to plaque formation and rupture. Oxidative stress and reduced nitric oxide contribute to vascular damage by impairing endothelium-mediated vasodilatation and promoting pro-thrombotic and pro-inflammatory states [5,6]. Additionally, 8-iso-PGF_2α_ directly induces vasoconstriction, platelet activation, and smooth muscle proliferation in the vessels [6,23], further exacerbating vascular damage. In hypertensive patients, oxidative stress is closely linked to inflammation and atherogenic activation, as oxidative excess reduces nitric oxide bioavailability and correlates with the degree of endothelium dysfunction and with subsequent cardiovascular events [3,11].

In summary, our study, in line with previous research, including studies on metabolic disorders [38], reinforces the association between 8-iso-PGF_2α_ and cardiovascular risk, underscoring the potential role of oxidative stress markers as valuable indicators for cardiovascular risk assessment across diverse patient populations. The study of inflammatory patterns could help in the better stratification of cardiovascular risk, thus potentially favoring target therapy; similarly, inflammatory molecules could be the target of therapies to reduce cardiovascular risk in the near future. However, further research, particularly prospective studies, is needed to confirm our findings and expand on their implications.

Our study has several limitations that should be considered when interpreting the results. The cross-sectional nature of the study prevents us from drawing conclusions about a causal relationship between 8-iso-PGF_2α_ and predicted cardiovascular risk. However, there are plausible mechanistic reasons to expect this, as described earlier. Regarding the measurement methodology, we quantified serum 8-iso-PGF_2α_, which likely reflects its levels only over short intervals. Additionally, the measurements were performed using ELISA, which, although widely used, is less sensitive and specific than mass spectrometry for assessing F_2_-isoprostanes. Measurements of IsoP using LCMS are costly, being limited to highly trained technicians and requiring expensive chromatography analysis and standards, which therefore limits its clinical utility. Lastly, the potential influence of confounding factors such as diet, systemic inflammation, and medication use on 8-iso-PGF_2α_ levels cannot be overlooked, as dietary patterns rich in antioxidants or pro-oxidant components, chronic inflammatory states, and specific pharmacological treatments—including lipid-lowering and anti-inflammatory drugs—may independently modulate oxidative stress markers and affect the observed associations [39,40,41]; therefore, further studies are needed to assess their impact and adjust for these variables in future analyses.

## 5. Conclusions

In conclusion, this study demonstrates for the first time that 8-iso-PGF_2α_, a reliable marker of oxidative stress, is independently associated with 10-year cardiovascular risk as predicted by validated models (Fr-S and ASCVD-S) in hypertensive patients without overt cardiovascular disease. This relationship was particularly evident in individuals with eGFR ≥ 60 mL/min/1.73 m^2^, highlighting a potential link between oxidative stress and early cardiovascular risk in this subgroup. Conversely, the absence of such an association in patients with impaired renal function suggests that advanced vascular damage in CKD may diminish the prognostic value of oxidative stress.

From a clinical perspective, these results underscore the importance of oxidative stress assessment in hypertension management, potentially aiding in personalized risk stratification and early preventive interventions. Given its independent association with cardiovascular risk, 8-iso-PGF_2α_ could complement existing risk scores, offering a more comprehensive evaluation of vascular health. However, additional longitudinal studies are required to establish a causal relationship between oxidative stress and cardiovascular outcomes and to investigate its potential therapeutic applications in both the general population and individuals with renal impairment.

## Figures and Tables

**Table 1:** 8-iso-PGF_2α_ in patients with low, moderate, or high cardiovascular risk calculated by the Framingham Risk Score or ASCVD Risk Score.

Variable *	Overall Population (*n* = 432)	eGFR ≥ 60 (*n* = 279)	eGFR < 60 (*n* = 153)	*p*-Value ^
Age (years)	60 ± 10	57 ± 10	65 ± 8	*<0.001*
Male sex, *n* (%)	255 (59)	173 (64.0)	82 (53.6)	*NS*
Smoking habit, *n* (%)	109 (25.3)	59 (21.15)	50 (32.8)	*NS*
Diabetes, *n* (%)	111 (25.7)	63 (22.6)	48 (31.4)	*NS*
Antihypertensive therapy, *n* (%)	415 (96.1)	270 (96.8)	145 (94.8)	*NS*
Clinic systolic BP (mmHg)	142 ± 21	143 ± 21	140 ± 20	*NS*
Clinic diastolic BP (mmHg)	84 ± 13	86±14	80 ± 11	*<0.001*
Clinic mean BP (mmHg)	103 ± 14	105 ± 15	100 ± 12	*0.001*
Clinic pulse pressure (mmHg)	58 ± 16	57 ± 15	60 ± 18	*NS*
Clinic heart rate (bpm)	73 ± 10	73 ± 10	72 ± 11	*NS*
Biochemical parameters				
Serum glucose (mg/dL)	110.1 ± 36.1	108.6 ± 31.7	112.8 ± 42.9	*NS*
Serum uric acid (mg/dL)	6.43 ± 1.65	6.39 ± 1.70	6.48 ± 1.59	*<0.001*
Serum total cholesterol (mg/dL)	191.5 ± 43.6	193.6 ± 40.3	187.6 ± 48.9	*NS*
LDL-c (mg/dL)	119.06 ± 38.80	121.69 ± 37.65	114.27 ± 40.32	*NS*
HDL-c (mg/dL)	46.11 ± 12.44	47.22 ± 11.79	44.10 ± 13.35	*<0.05*
Serum triglycerides (mg/dL)	118 (86–161)	105 (81–152)	136 (104–177)	*<0.001*
Serum creatinine (mg/dL)	1.43 ± 1.14	0.92 ± 0.16	2.36 ± 1.53	*<0.001*
eGFR (ml/min/1.73 m^2^)	65.9 ± 27.5	83.5 ± 12.8	33.8 ± 16.1	*<0.001*
Serum sodium (mEq/L)	139 ± 3	140 ± 3	139 ± 3	*NS*
Serum potassium (mEq/L)	4.35 ± 0.40	4.33 ± 0.38	4.37 ± 0.43	*NS*
Endothelial disfunctions and cardiovascular risk				
8-iso-PGF_2α_ (pg/mL)	292.6 ± 125.7	247.2 ± 104.7	375.4 ± 118.7	*<0.001*
CRP (mg/dL)	2.40 (1.60–3.30)	2.00 (1.39–2.70)	3.17 (2.40–3.80)	*<0.001*
Framingham Risk Score (%)	7.46 (4.17–14.06)	6.49 (3.60–11.76)	9.44 (6.00–17.83)	*0.001*
Framingham Risk Score < 10%, *n* (%)	272 (63.0)	193 (69.2)	79 (51.6)	*<0.001*
Framingham Risk Score ≥ 20%, *n* (%)	61 (14.1)	36 (12.9)	25 (16.3)	*NS*
ASCVD Risk Score (%)	10.92 (4.92–21.43)	8.25 (4.24–17.28)	15.83 (9.59–28.27)	*<0.001*
ASCVD Risk Score < 7.5%, *n* (%)	157 (36.3)	129 (46.2)	28 (18.3)	*<0.001*
ASCVD Risk Score ≥ 15%, *n* (%)	167 (38.7)	87 (31.2)	80 (52.3)	*<0.001*

* Continuous variables are reported as either mean ± standard deviation or median with interquartile range, based on their distribution. ^ Comparison between eGFR-based groups; non-significant (NS): *p* > 0.05. Abbreviations: eGFR—estimated glomerular filtration rate; BP—blood pressure; LDL-c—low-density lipoprotein cholesterol; HDL-c—high-density lipoprotein cholesterol; 8-iso-PGF_2α_—8-iso-prostaglandin F_2α_; CRP—C-reactive protein; ASCVD—atherosclerotic cardiovascular disease.

**Table 2:** Main correlations of 8-iso-PGF_2α_ and cardiovascular risk scores with other variables in the entire study population.

	8-Iso-PGF_2α_	Framingham Risk Score	ASCVD Risk Score
	*r*	*r*	*r*
Age (years)	0.383 ***	0.778 ***	0.859 ***
Serum glucose (mg/dL)	0.202 ***	0.377 ***	0.345 ***
Serum uric acid (mg/dL)	−0.051 *^NS^*	0.234 ***	0.273 ***
Serum total cholesterol (mg/dL)	−0.131 **	−0.301 ***	−0.160 ***
LDL-c (mg/dL)	−0.165 ***	−0.090 *	−0.156 **
HDL-c (mg/dL)	−0.027 *^NS^*	−0.288 ***	−0.256 ***
Serum triglycerides (mg/dL)	0.090 *^NS^*	0.088 *^NS^*	0.147 **
Serum creatinine (mg/dL)	0.466 ***	0.127 **	0.177 ***
eGFR (mL/min/1.73 m^2^)	−0.520 ***	−0.254 ***	−0.338 ***
Serum sodium (mEq/L)	−0.024 *^NS^*	−0.085 *^NS^*	−0.009 *^NS^*
Serum potassium (mEq/L)	0.086 *^NS^*	0.088 *^NS^*	0.084 *^NS^*
Systolic BP (mmHg)	0.188 ***	0.236 ***	0.156 ***
Diastolic BP (mmHg)	−0.015 *^NS^*	−0.163 ***	−0.247 ***
Mean BP (mmHg)	0.083 *^NS^*	0.014 *^NS^*	−0.076 *^NS^*
Pulse Pressure (mmHg)	0.250 ***	0.430 ***	0.395 ***
Heart Rate (bpm)	−0.046 *^NS^*	−0.074 *^NS^*	−0.094 *
CRP (mg/dL)	0.717 ***	0.407 ***	0.404 ***

***: *p* ≤ 0.001; **: *p* ≤ 0.01; *: *p* ≤ 0.05; *NS*: *p* > 0.05. Abbreviations: ASCVD—atherosclerotic cardiovascular disease; LDL-c—low-density lipoprotein cholesterol; HDL-c—high-density lipoprotein cholesterol; eGFR—estimated glomerular filtration rate; BP—blood pressure; 8-iso-PGF_2α_—8-iso-prostaglandin F_2α_; CRP—C-reactive protein.

**Table 3:** Independent multivariate correlates of Framingham Risk Score [A] and ASCVD Risk Score [B] in the overall study population.

**[A]** **Outcome Variable:** **Framingham Risk Score**	**Regression Coefficients**			
	**Standardized**			
	**Β**	**β**	**t**	***p*-Value**
Model (R^2^ = 0.938)				
Age	0.024	0.683	45.810	<0.001
Diabetes	0.274	0.326	24.988	<0.001
Systolic BP	0.005	0.277	21.928	<0.001
Sex (male)	0.178	0.240	18.354	<0.001
Smoking habit	0.166	0.165	12.780	<0.001
HDL cholesterol	0.002	0.079	5.844	<0.001
Serum total cholesterol	0.001	−0.059	−4.357	0.001
eGFR	<0.001	0.066	4.128	0.001
8-iso-PGF_2α_	<0.001	0.052	3.236	0.001
Constant	−1.582	-	−27.712	<0.001
**[B]** **Outcome Variable:** **ASCVD Risk Score**	**Regression Coefficients**			
	**Standardized**			
	**Β**	**β**	**t**	***p*-Value**
Model (R^2^ = 0.969)				
Age	0.038	0.891	82.357	<0.001
Diabetes	0.245	0.244	26.431	<0.001
Sex (male)	0.207	0.232	25.019	<0.001
Systolic BP	0.005	0.216	24.131	<0.001
Serum total cholesterol	0.002	0.177	18.455	<0.001
HDL cholesterol	−0.006	−0.168	−17.513	<0.001
Smoking habit	0.072	0.060	6.562	<0.001
Antihypertensive therapy	0.129	0.057	6.482	<0.001
eGFR	<0.001	0.036	3.150	0.002
8-iso-PGF_2α_	<0.001	0.026	2.285	0.023
Constant	−2.384	-	−43.679	<0.001

Abbreviations: BP—blood pressure; HDL—high-density lipoprotein cholesterol; eGFR—estimated glomerular filtration rate; 8-iso-PGF_2α_—8-iso-prostaglandin F_2α_; ASCVD—atherosclerotic cardiovascular disease. B—unstandardized coefficient, β—standardized regression coefficient, t—t-value, R^2^—coefficient of determination.

## Data Availability

Data are available on reasonable request.

## Supplementary Materials

The following supporting information can be downloaded at: https://www.mdpi.com/article/10.3390/life15030401/s1. Figure S1: Patient criteria.
