# Supplementary material for: Relationship Between 8-iso-prostaglandin-F2α and Predicted 10-Year Cardiovascular Risk in Hypertensive Patients
Source: Life (Basel). 2025 Mar 4;15(3):401. doi: 10.3390/life15030401 (PMC11943662; doi:10.3390/life15030401)
Supplement: Supplementary file 1 [file life-15-00401-s001.zip › life-3482205-supplementary.pdf]

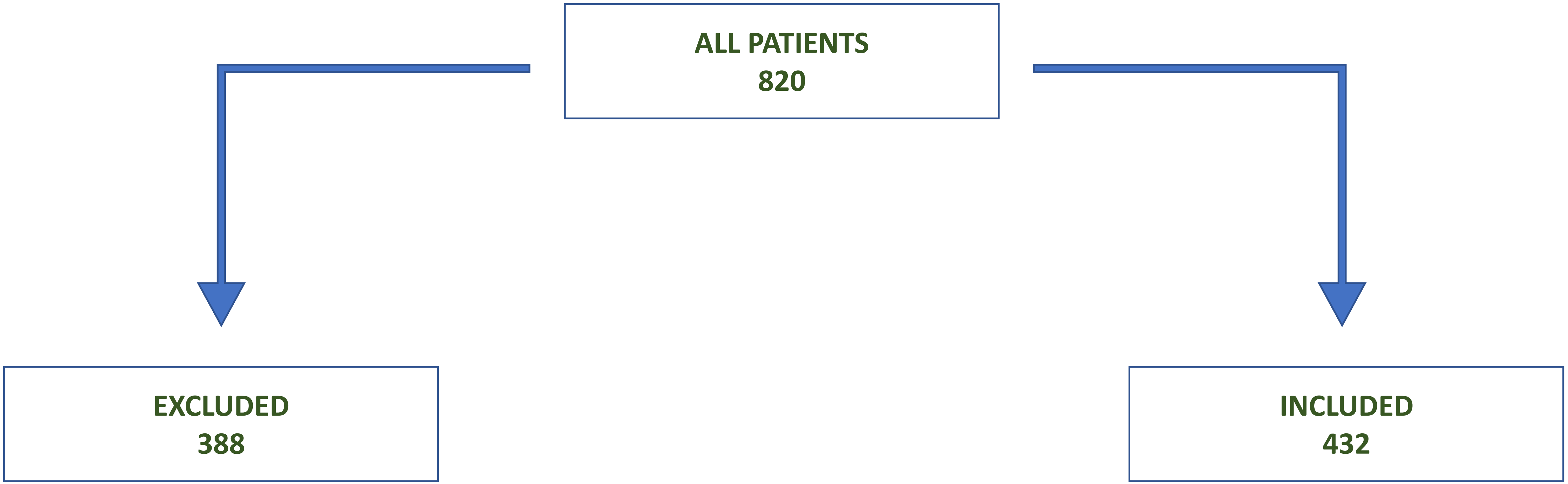

- **175 patients -** Age < 40 and > 75 years old.
- **116 patients -** History of cerebrovascular disease, coronary heart disease, or symptomatic peripheral arterial disease
- **97 patients -** Other causes
